# Overexpression of Human ABCB1 and ABCG2 Reduces the Susceptibility of Cancer Cells to the Histone Deacetylase 6-Specific Inhibitor Citarinostat

**DOI:** 10.3390/ijms22052592

**Published:** 2021-03-05

**Authors:** Chung-Pu Wu, Cheng-Yu Hung, Sabrina Lusvarghi, Yen-Fu Chang, Sung-Han Hsiao, Yang-Hui Huang, Tai-Ho Hung, Jau-Song Yu, Suresh. V. Ambudkar

**Affiliations:** 1 Graduate Institute of Biomedical Sciences, College of Medicine, Chang Gung University, Tao-Yuan 333, Taiwan; book269874522@gmail.com (Y.-F.C.); johnson170_ya@hotmail.com (S.-H.H.); yanghui.huang01@gmail.com (Y.-H.H.); yusong@mail.cgu.edu.tw (J.-S.Y.); 2 Department of Physiology and Pharmacology, College of Medicine, Chang Gung University, Tao-Yuan 333, Taiwan; 3 Molecular Medicine Research Center, College of Medicine, Chang Gung University, Tao-Yuan 333, Taiwan; aruhung@gmail.com; 4 Department of Obstetrics and Gynecology, Taipei Chang Gung Memorial Hospital, Taipei 10507, Taiwan; thh20@adm.cgmh.org.tw; 5 Laboratory of Cell Biology, CCR, NCI, NIH, Bethesda, MD 20892, USA; sabrina.lusvarghi@nih.gov (S.L.); ambudkar@mail.nih.gov (S.V.A.); 6 Department of Chinese Medicine, College of Medicine, Chang Gung University, Tao-Yuan 333, Taiwan; 7 Department of Biochemistry and Molecular Biology, College of Medicine, Chang Gung University, Tao-Yuan 333, Taiwan; 8 Liver Research Center, Chang Gung Memorial Hospital, Linkou, Tao-Yuan 333, Taiwan

**Keywords:** ABCB1, ABCG2, multidrug resistance, HDAC6, citarinostat

## Abstract

Citarinostat (ACY-241) is a promising oral histone deacetylase 6 (HDAC6)-selective inhibitor currently in clinical trials for the treatment of multiple myeloma (MM) and non-small-cell lung cancer (NSCLC). However, the inevitable emergence of resistance to citarinostat may reduce its clinical effectiveness in cancer patients and limit its clinical usefulness in the future. In this study, we investigated the potential role of the multidrug efflux transporters ABCB1 and ABCG2, which are two of the most common mechanisms of acquired resistance to anticancer drugs, on the efficacy of citarinostat in human cancer cells. We discovered that the overexpression of ABCB1 or ABCG2 significantly reduced the sensitivity of human cancer cells to citarinostat. We demonstrated that the intracellular accumulation of citarinostat and its activity against HDAC6 were substantially reduced by the drug transport function of ABCB1 and ABCG2, which could be restored by treatment with an established inhibitor of ABCB1 or ABCG2, respectively. In conclusion, our results revealed a novel mechanism by which ABCB1 and ABCG2 actively transport citarinostat away from targeting HDAC6 in cancer cells. Our results suggest that the co-administration of citarinostat with a non-toxic modulator of ABCB1 and ABCG2 may optimize its therapeutic application in the clinic.

## 1. Introduction

Histone deacetylases (HDACs) are a family of enzymes that remove acetyl groups from the acetylated histone and non-histone proteins, leading to chromatin condensation and transcriptional repression [1]. Many important cellular processes such as transcriptional regulation, cell cycle control, DNA damage repair, apoptosis, and autophagy are regulated by HDACs [2]. Consequently, the dysregulation of HDAC activity can cause abnormal gene expression and cell signaling that promote tumor cell initiation and proliferation, making the HDACs promising therapeutic targets for drug discovery [2,3,4]. Non-selective pan-HDAC inhibitors panobinostat (LBH589) and belinostat (PXD101) have been approved by the U.S. Food and Drug Administration (FDA) for use in patients with multiple myeloma (MM) [5] and relapsed or refractory peripheral T-cell lymphoma [6], respectively. However, unpredicted adverse events have been reported in patients receiving these pan-HDAC inhibitors presumably due to the lack of selectivity [7,8,9,10,11,12,13]. Alternatively, researchers have been developing isotype-selective HDAC inhibitors, such as ricolinostat (ACY-1215), that target class IIb HDAC relative to class I HDACs [14,15,16,17,18,19,20,21]. The class IIb HDAC, HDAC6, is localized predominantly within the cytoplasm and deacetylates non-histone substrates such as α-tubulin and heat shock protein 90 (Hsp90) [22,23,24].

Citarinostat (ACY-241) is a second-generation, bioavailable, and selective inhibitor of class IIb histone deacetylase 6 (HDAC6) [20] that is active against MM [10,21] and acts synergistically in combination with other therapeutic agents against solid tumors and blood cancers [25,26,27,28,29]. Citarinostat is currently being evaluated in clinical trials, both alone and in combination with other therapeutic agents, for patients with advanced solid tumors (ClinicalTrials.gov Identifier: NCT02551185), unresectable non-small cell lung cancer (NSCLC)(NCT02635061), relapsed or relapsed-and-refractory MM (NCT02400242), smoldering MM (SMM) (NCT02886065), and unresectable Stage III/Stage IV melanoma (NCT02935790). Although citarinostat demonstrates promising results in early studies, the inevitable emergence of resistance to citarinostat is most likely to present a significant therapeutic challenge and limit its clinical usefulness. Therefore, understanding the potential mechanism of citarinostat resistance is crucial in the development of an appropriate therapeutic strategy to prolong the effectiveness of this drug in the future.

The ATP-binding cassette (ABC) drug transporters ABCB1 (MDR1; P-glycoprotein) and ABCG2 (BCRP; MXR) are transport proteins that translocate a large diversity of conventional and molecularly targeted anticancer drugs using energy derived from ATP binding and hydrolysis [30,31,32,33,34]. Consequently, ABCB1 and ABCG2 are often associated with the development of multidrug resistance (MDR) in human cancer cells that could result in relapse and treatment failure in cancer patients [34,35,36]. High expression of ABCB1 and ABCG2 has been associated with reduced chemosensitivity and MDR phenotype in blood cancers such as MM [37,38,39,40,41,42], chronic lymphocytic leukemia (CLL) [43], acute lymphocytic leukemia (ALL), and acute myelogenous leukemia (AML) [44,45,46], as well as solid tumors such as metastatic breast cancer [47]. Furthermore, ABCB1 and ABCG2 are highly expressed in the intestinal epithelium, liver hepatocytes, the blood–placenta barrier (BPB), and the blood–brain barrier (BBB), limiting the oral availability, tissue distribution, and the efficacy of many therapeutic agents [34,48]. Therefore, it is important to determine the potential interaction between citarinostat and these two multidrug efflux transporters.

We have discovered previously that the efficacy of ricolinostat (ACY-1215), a first-in-class orally available inhibitor of HDAC6, is significantly compromised in human cancer cells. Therefore, the aim of this study was to investigate the impact of ABCB1 and ABCG2, two of the most common mechanisms of resistance to conventional and molecularly targeted anticancer drugs [49,50,51,52,53,54], on the efficacy of citarinostat in human cancer cell lines. Our data demonstrated that the intracellular concentration of citarinostat and the HDAC6 inhibiting activity were significantly reduced by ABCB1 and ABCG2, and consequently, cancer cells overexpressing ABCB1 or ABCG2 are insensitive to citarinostat. Taken together, our study revealed that the drug transport mediated by ABCB1 or ABCG2 represents a novel mechanism for acquired resistance to citarinostat in human cancer cells; therefore, combination therapies will need to be tested to overcome this clinical problem in the future.

## 2. Results

### 2.1. Citarinostat Is Less Cytotoxic in Cells Overexpressing ABCB1 or ABCG2

**Figure 1:**
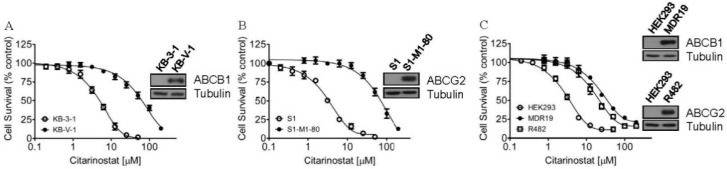
Citarinostat is less cytotoxic in cells overexpressing ABCB1 or ABCG2. (**A**) Parental KB-3-1 cells (open circles) and ABCB1-overexpressing KB-V-1 cells (filled circles); (**B**) parental S1 cells (open circles) and ABCG2-overexpressing S1-M1-80 cells (filled circles); as well as (**C**) parental pcDNA-HEK293 cells (open circles), ABCB1-transfected MDR19-HEK293 cells (filled circles), and ABCG2-transfected R482-HEK293 (open squares) cells, were treated with increasing concentrations of citarinostat for 72 h and processed as described previously [52]. The representative immunoblots of ABCB1, ABCG2, and tubulin as a loading control in drug-sensitive cells and multidrug-resistant cells are shown (*inset*). *Points*, mean values from at least three independent experiments; *bars*, SEM.

To examine the effect of ABCB1 and ABCG2 on the chemosensitivity of human cancer cell lines to citarinostat, we determined the cytotoxicity of citarinostat in multiple pairs of ABCB1- or ABCG2-overexpressing multidrug-resistant cancer cell lines and the respective drug-sensitive parental lines. We first noticed that citarinostat was significantly more cytotoxic to the parental human KB-3-1 epidermal cancer cells and the parental human S1 colon cancer cells than to the respective ABCB1-overexpressing variant KB-V-1 (R.F = 15, Figure 1A) and ABCG2-overexpressing variant S1-M1-80 (R.F = 21, Figure 1B) cells. Similarly, as summarized in Table 1, the parental human OVCAR-8 ovarian cancer cells and human H460 lung cancer cells were also significantly more sensitive to citarinostat as compared to the respective ABCB1-overexpressing variant NCI-ADR-RES (R.F = 11) and ABCG2-overexpressing variants H460-MX20 (R.F = 3). To verify our observation, we examined the cytotoxicity of citarinostat in HEK293 cells and HEK293 cells transfected with either human ABCB1 (MDR19-HEK293) or human ABCG2 (R482-HEK293). The fact that MDR19-HEK293 and R482-HEK293 cells were 11-fold and 7-fold more resistant to citarinostat as compared to the parental HEK293 cells supports our finding that ABCB1- and ABCG2-overexpressing cells are resistant to citarinostat (Figure 1C). More importantly, we found that the chemosensitivity of citarinostat in cells overexpressing ABCB1 or ABCG2 could be completely restored by tariquidar or Ko143 at 1 µM (Table 1). Tariquidar is a specific ABCB1 inhibitor, whereas Ko143 is a specific ABCG2 inhibitor. These results suggest that ABCB1- and ABCG2-mediated efflux of citarinostat contributes directly to citarinostat resistance in multidrug-resistant cancer cells.

### 2.2. The Effect of Citarinostat on the Activity of HDAC6 Is Reduced by ABCB1 and ABCG2 in Human Cancer Cell Lines

**Figure 2:**
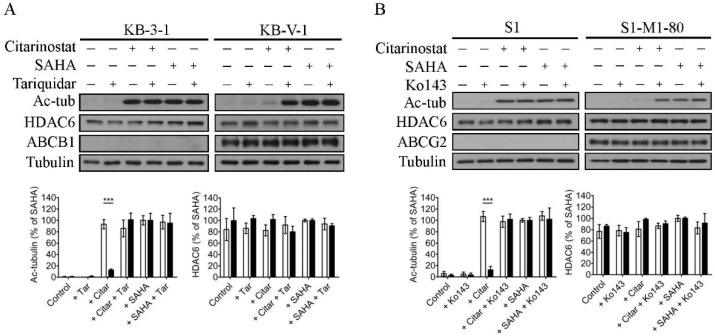
Inhibition of HDAC6-mediated α-tubulin deacetylation by citarinostat. Representative immunoblots of acetylated α-tubulin (Ac-tub), total HDAC6, ABCB1 or ABCG2, and total tubulin in (**A**) human KB-3-1 epidermal carcinoma cell line and its ABCB1-overexpressing multidrug-resistant variant KB-V-1, as well as in (**B**) human S1 colon carcinoma cell line and its ABCG2-overexpressing multidrug-resistant variant S1-M1-80 are shown. Cells were treated with DMSO, 1 µM of citarinostat, or 25 µM of a known HDAC inhibitor SAHA as a positive control, in the presence or absence of 1 µM of an ABCB1 reference inhibitor tariquidar or an ABCG2 reference inhibitor Ko143 for 2 h at 37 °C before immunoblotting. Quantification of Ac-tub and HDAC6 in KB-3-1 (**A**, empty bars), KB-V-1 (**A**, filled bars) cells, S1 (**B**, empty bars), and S1-M1-80 (**B**, filled bars) cells are presented as mean ± S.D. calculated from at least three independent experiments. *** *p* < 0.001, versus the same treatment in parental cells.

Previous reports have demonstrated that citarinostat characteristically inhibits the deacetylase activity of HDAC6 and induces apoptosis in human cancer cell lines [20,21]. To this end, we investigated the effect of ABCB1 and ABCG2 on the efficacy of citarinostat in human cancer cells by examining the acetylation level of α-tubulin (Ac-tub), which is a known non-histone substrate of HDAC6 [22], in drug-sensitive cancer cell lines and multidrug-resistant variants overexpressing ABCB1 or ABCG2. As shown in Figure 2, KB-3-1, KB-V-1, S1, and S1-M1-80 cells were treated with DMSO (control), 1 µM of citarinostat, or 25 µM of suberoylanilide hydroxamic acid (SAHA), in the presence or absence of 1 µM of tariquidar or Ko143 as indicated. Of note, a high concentration of SAHA, a known HDAC inhibitor, was used here as a control [55]. As expected, α-tubulin was typically deacetylated by the tubulin deacetylase HDAC6, and citarinostat promotes α-tubulin hyperacetylation in cancer cells by inhibiting the activity of HDAC6. However, we found that citarinostat had a significantly reduced effect on HDAC6 in ABCB1-overexpressing KB-V-1 (Figure 2A, right panels) and ABCG2-overexpressing S1-M1-80 (Figure 2B, right panels) cancer cells as compared to the respective parental KB-3-1 (Figure 2A, left panels) and S1 (Figure 2B, left panels) cancer cells. More importantly, the extent of α-tubulin acetylation induced by citarinostat in KB-V-1 and S1-M1-80 cancer cells was completely restored by tariquidar and Ko143, respectively (Supplementary Figure S1). Of note, the level of HDAC6, ABCB1, ABCG2, and total tubulin remained constant in all cell lines.

**Figure 3:**
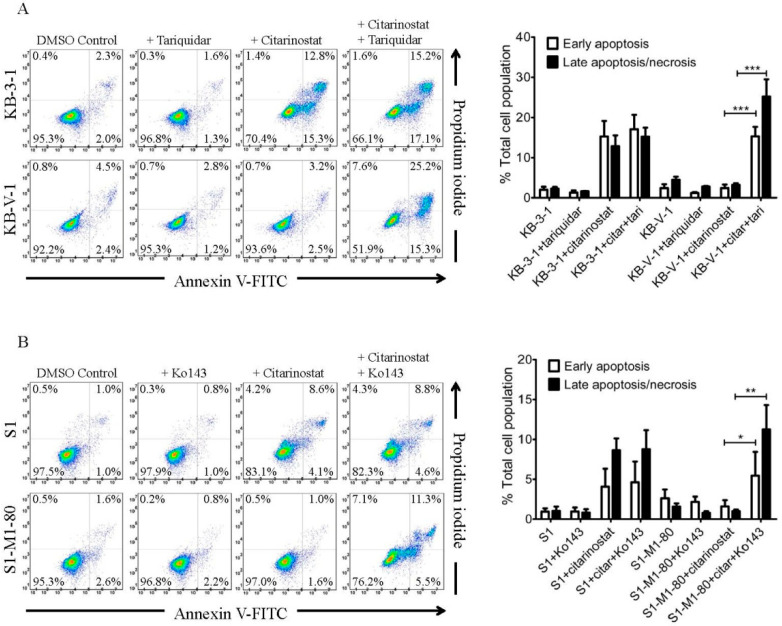
Citarinostat induces apoptosis in drug-sensitive cancer cell lines. (**A**) KB-3-1 and KB-V-1 cells were treated with DMSO (control), 1 µM of tariquidar, 1 µM of citarinostat, or a combination of 1 µM of citarinostat and tariquidar, whereas (**B**) S1 and S1-M1-80 cells were treated with DMSO (control), 1 µM of Ko143, 1 µM of citarinostat, or a combination of 1 µM of citarinostat and Ko143 as indicated. Cells were subsequently processed using the Annexin V-Fluorescein-5-isothiocyanate (FITC) and propidium iodide (PI) staining method and analyzed by flow cytometry as described previously [52]. Representative dot plots (left panels) and quantified values (right panels) are mean values ± S.D. calculated from three independent experiments. * *p* < 0.05; ** *p* < 0.01; *** *p* < 0.001, versus the treatment with tariquidar or Ko143.

Next, we determined the effect of tariquidar and Ko143 on citarinostat-induced apoptosis in the same cancer cell lines. As shown in Figure 3, citarinostat substantially increased the apoptotic cell population in KB-3-1 (from a basal level of approximately 4% to 28%) and S1 (from a basal level of approximately 2% to 13%) cancer cells, but not in KB-V-1 (Figure 3A) and S1-M1-80 (Figure 3B) cancer cells. Moreover, without directly inducing apoptosis themselves, tariquidar and Ko143 significantly increased the citarinostat-induced apoptotic cell population in KB-V-1 cells (from a basal level of approximately 4% to 40%) and S1-M1-80 cells (from a basal level of approximately 3% to 17%), respectively (Figure 3). These results are in agreement with the cytotoxicity data (Table 1) that the efficacy of citarinostat was significantly reduced by ABCB1 and ABCG2 in human cancer cell lines.

### 2.3. The Intracellular Accumulation of Citarinostat Is Reduced by ABCB1 and ABCG2 in Human Cancer Cell Lines

**Figure 4:**
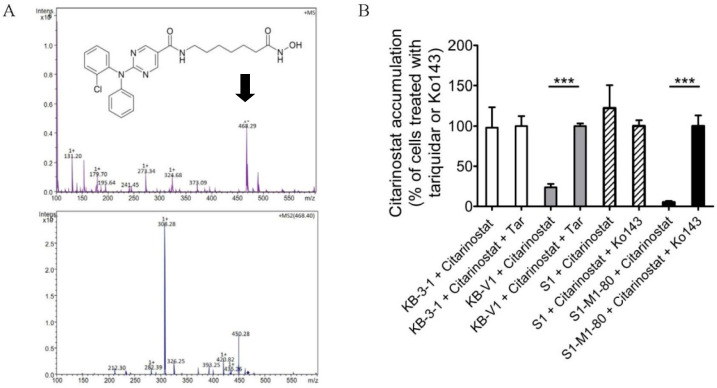
The intracellular concentration of citarinostat (molecular weight, 467.9 g/mol) is reduced by the drug transport activity of ABCB1 and ABCG2 in human cancer cells. (**A**) The chemical structure and product ion mass spectra of citarinostat. (**B**) KB-3-1 (white bars), KB-V-1 (gray bars), S1 (striped bars), and S1-M1-80 (black bars) cells were treated with 10 μM of citarinostat in the presence or absence of 10 μM of tariquidar or Ko143 at 37 °C for 60 min. The intracellular accumulation of citarinostat was quantified as described in Materials and Methods. Values are presented as mean values ± S.D. calculated from three independent experiments. *** *p* < 0.001, versus the treatment with tariquidar or Ko143.

One of the most probable explanations for reduced drug efficacy in ABCB1- and ABCG2-overexpressing multidrug-resistant cancer cells is the reduced intracellular drug accumulation caused by ABCB1- and ABCG2-mediated drug efflux [34]. To this end, we treated KB-3-1, KB-V-1, S1, and S1-M1-80 cells with citarinostat in the presence or absence of tariquidar or Ko143 and determined the intracellular concentration of citarinostat in these cells using a liquid chromatography/tandem mass spectrometry (LC-MS/MS) method as described previously [56,57] (Figure 4A). We found that the reduced intracellular accumulation of citarinostat in KB-V-1 and S1-M1-80 cancer cells (Figure 4B) was significantly restored by tariquidar and Ko143, signifying that the activity of ABCB1 and ABCG2 contributes greatly to the reduced efficacy of citarinostat in these cancer cells.

### 2.4. Docking of Citarinostat in the Drug-Binding Pocket of ABCB1 and ABCG2

**Figure 5:**
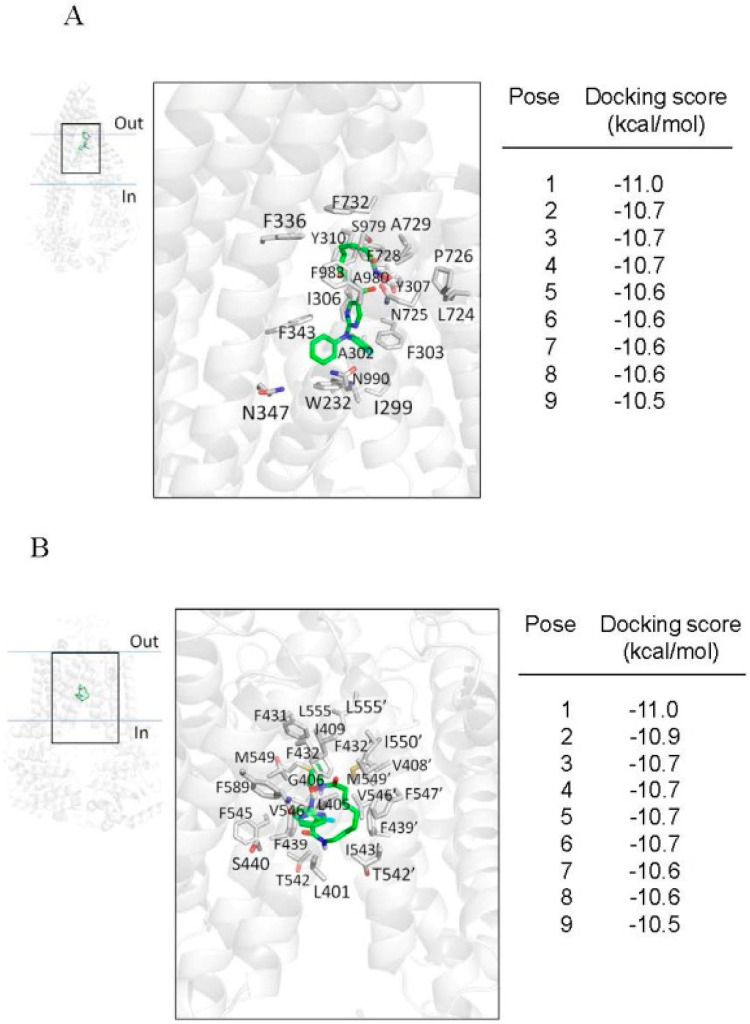
Docking of citarinostat with atomic structure of ABCB1 and ABCG2. The lowest energy poses for binding of citarinostat within the drug-binding region of (**A**) ABCB1 (PDB: 6QEX) and (**B**) ABCG2 (PDB:5NJG) were obtained after exhaustive docking using AutoDock Vina software as described in Materials and Methods. The lowest energy pose (pose 1) for citarinostat in the drug-binding pocket of ABCB1 and ABCG2 is presented to illustrate the residues that are within 4Å of the ligand citarinostat (shown in gray). Figures were prepared using PyMOL molecular graphics system, Version 1.7 Schrödinger, LLC.

Next, the in silico molecular docking analysis of the lowest energy docking poses of citarinostat in the inward-open structure of human ABCB1 (PDBID:6QEX) [58] and ABCG2 (PDBID:5NJ3) [59] was performed to gain insight into the interactions between citarinostat and the substrate-binding pocket in the transmembrane domains of ABCB1 and ABCG2. A docking grid for each transporter was defined to include all the residues that comprise the binding cavity. The side chains of residues previously shown to interact with other substrates or modulators were set as flexible to maximize all possible interactions with the citarinostat. The docking simulation provided the nine lowest energy poses, whose energy (in kcal/mol) is presented in Figure 5 in the right panels. From these nine poses, we chose the lowest energy pose for further graphical analysis.

The interactions between citarinostat and residues located within the drug-binding pocket of ABCB1 and ABCG2 are shown in Figure 5A,B, respectively. In both cases, the lowest energy pose (−11.0 kcal/mol) depicts the citarinostat molecule binding in the binding cavity of each transporter with extensive aromatic and hydrophobic interactions with nearby amino acids, particularly with residues of transmembrane helices 5–7 and 12 of ABCB1 and with helices 1,5,6 of both monomers of ABCG2.

## 3. Discussion

The dysregulation of HDAC activity is an epigenetic hallmark of malignancies such as multiple myeloma [60]. Consequently, HDAC has been considered by many as an important therapeutic target, and the development of novel inhibitors targeting HDAC activity has become an increasingly important area of drug development in recent years [61,62]. Citarinostat is currently being evaluated in clinical trials, both as a single agent and combination therapy, for patients with advanced solid tumors, unresectable NSCLC, unresectable Stage III/Stage IV melanoma, relapsed or relapsed-and-refractory MM, and SMM. Recently, the potential benefit of combining citarinostat with conventional anticancer drugs in cancer treatment has been investigated extensively. Huang et al. demonstrated that the combination treatment of paclitaxel with citarinostat significantly suppresses solid tumor growth in cell lines and xenograft models [20]. Moreover, citarinostat synergizes pomalidomide, a thalidomide-based immunomodulatory drug, in in vitro assays, and in vivo murine xenograft model [21]. Ray et al. demonstrated that the combination of citarinostat with anti-programmed death ligand-1 (PD-L1) antibody enhances anti-tumor immunity and cytotoxicity in multiple myeloma [25], whereas the combination of Alpha-Enolase (ENO1) inhibitor and citarinostat enhances autologous MM-specific CD8+ cytotoxic T lymphocyte activity [26]. More recently, a study found that the combination treatment of citarinostat and momelotinib, an inhibitor of Janus kinase/signal transducer of transcription-3 (JAK/STAT3) signaling [27], improved efficacy in lymphoid malignant cell lines. Cho et al. demonstrated that the combined treatment of citarinostat and the bromodomain and extra-terminal (BET) domain inhibitor JQ1 synergistically suppresses metastasis of head and neck squamous cell carcinoma (HNSCC) via desregulating matrix metalloproteinase (MMP)-2, MMP-9, and MMP-14 [29]. Furthermore, a recent study reported that the HDAC6 inhibitors ricolinostat and citarinostat could upregulate CD38 expression in MM cells and augment the efficacy of the anti-CD38 antibody daratumumab [28]. Clinical trials data have shown that citarinostat is well-tolerated as monotherapy and in combination with pomalidomide and dexamethasone [10]. However, despite these results, the potential mechanism of acquired resistance to citarinostat in cancer cells, which could present a therapeutic challenge in the future, remains unclear.

Previous studies have reported that the expression of ABCB1 and ABCG2 is often upregulated by treatment with anticancer drugs in most MM patients [39,40,42]. The relationship between HDAC6 and ABCB1 was reported by Cheng et al., in which HDAC6 could upregulate the expression of ABCB1 through the upregulation of interleukin (IL)-8, causing significant drug resistance to doxorubicin in osteosarcoma cells [63]. Moreover, we discovered that both ABCB1 and ABCG2 confer significant resistance to ricolinostat [56], which is the first reported selective HDAC6 inhibitor that is structurally similar to citarinostat. Therefore, we investigated whether the activity of citarinostat is also affected by the drug efflux function of ABCB1 and ABCG2 in human cancer cell lines in a similar manner as ricolinostat. Upon examining the effect of ABCB1 and ABCG2 on the toxicity and efficacy of citarinostat in human cancer cell lines, we found that the proliferation of cancer cell lines, regardless of the tissue of origin, was inhibited by citarinostat with the IC_50_ value ranging from 3 to 8 μM, which is similar to the value reported previously by Huang et al. [20] and North et al. [21]. However, we noticed that the IC_50_ value of citarinostat in ABCB1- and ABCG2-overexpressing cancer cells was significantly higher than the value of citarinostat in the respective drug-sensitive parental cancer cells, indicating that these multidrug-resistant cancer cells are resistant to citarinostat (Table 1). Furthermore, the fact that HEK293 cells with ectopic expression of human ABCB1 or human ABCG2 were also resistant to citarinostat verified our findings. In addition to determining the cytotoxicity, we examined the inhibitory effect of citarinostat on HDAC6 activity by determining the level of acetylated tubulin in cell lines overexpressing ABCB1 or ABCG2. We found that the efficacy of citarinostat on attenuating the HDAC6 deacetylase activity (Figure 2) and the induction of apoptosis (Figure 3) correspond directly to the reduced accumulation of citarinostat in ABCB1- and ABCG2-overexpressing human cancer cells (Figure 4). More importantly, by blocking the drug-efflux function of ABCB1 and ABCG2, tariquidar and Ko143 increased the intracellular concentration of citarinostat significantly, and they consequently restored the cytotoxicity and the efficacy of citarinostat to inhibit HDAC6 in ABCB1- and ABCG2-overexpressing multidrug-resistant cancer cells. The in silico molecular docking analysis of citarinostat with the inward-open structure of human ABCB1 (PDBID:6QEX) [58] and ABCG2 (PDBID:5NJ3) provided additional information on the binding of citarinostat within the substrate-binding pockets of human ABCB1 and ABCG2. We found that citarinostat had an outstanding docking score for binding to the drug-biding pocket in the transmembrane region of both transporters. Specific hydrophobic and aromatic interactions between citarinostat and nearby residues in the pocket were identified. The binding energy score suggests a strong affinity of citarinostat for both transporters similar to other modulators (refs [56,57]). Collectively, the results of this study support the conclusion that citarinostat is a substrate for both ABCB1 and ABCG2. However, the effect of prolonged citarinostat exposure on the protein expression of ABCB1 and ABCG2 in cancer cells remains to be determined.

## 4. Materials and Methods

### 4.1. Chemicals

Dulbecco’s Modified Eagle’s Medium (DMEM), RPMI-1640 medium, fetal calf serum (FCS), phosphate-buffered saline (PBS), trypsin-ethylenediaminetetraacetic acid (EDTA), l-glutamine, penicillin, and streptomycin were purchased from Gibco, Invitrogen (Carlsbad, CA, USA). An Annexin V:FITC Apoptosis Detection Kit was purchased from BD Pharmingen (San Diego, CA, USA). A Tools Cell Counting Kit (CCK-8) was purchased from Biotools Co., Ltd. (Taipei, Taiwan). Tariquidar, Ko143, and all other chemicals were purchased from Sigma-Aldrich (St. Louis, MO, USA). Citarinostat (ACY-241) was purchased from Selleckchem (Houston, TX, USA).

### 4.2. Cell Lines and Culture Conditions

Parental KB-3-1 human epidermal carcinoma cells, the ABCB1-overexpressing KB-V-1 cells, parental OVCAR-8 human ovarian carcinoma cells, and ABCB1-overexpressing NCI-ADR-RES cells; Empty vector (pcDNA3.1), ABCB1-transfected (MDR19), and ABCG2-transfected (R482) human embryonic kidney (HEK293) cells were maintained in DMEM supplemented with 10% FCS, 2 mM l-glutamine, and 100 units of penicillin/streptomycin/mL. KB-V-1 cells were cultured in the presence of 1 μg/mL vinblastine [64]; NCI-ADR-RES cells were cultured in the presence of 0.85 µM doxorubicin [65]. HEK293 transfected lines were maintained in 2 mg/mL G418, as described previously [66]. Parental H460 human non-small cell lung cancer (NSCLC) cells and ABCG2-overexpressing H460-MX20 cells; parental S1 human colon carcinoma cells and ABCG2-overexpressing S1-M1-80 cells were maintained in RPMI-1640 medium supplemented with 10% FCS, 2 mM l-glutamine, and 100 units of penicillin/streptomycin/mL. S1-M1-80 cells were cultured in the presence of 80 μM of mitoxantrone [67]; H460-MX20 cells were cultured in the presence of 20 nM of mitoxantrone [68]. All cell lines were maintained at 37 °C in 5% CO_2_ humidified air and placed in drug-free medium 7 days prior to assay.

### 4.3. Cytotoxicity Assays

Cytotoxicity assays were performed based on the method reported by Ishiyama et al. [69]. Briefly, cells were plated into 96-well plates at a density of 5000 cells/well in culture medium at 37 °C in 5% CO_2_ humidified air for 24 h for cells to attach. Cells were treated with citarinostat or drug combinations at varying concentrations with 0.5% (*v*/*v*) final concentration of DMSO in all wells for an additional 72 h in 5% CO_2_ humidified air at 37 °C. Plates were subsequently developed using MTT (3-(4,5-dimethylthiazol-2-yl)-2,5-diphenyl tetrazolium bromide) or CCK-8 reagents as described previously [53].

### 4.4. Immunoblot

An immunoblot assay using antibodies C219 (#517310, Merck Millipore, Burlington, Massachusetts, USA) at 1:3000, BXP-21 (#ab3380, Abcam, Cambridge, MA, USA) at 1:15,000, anti-HDAC6 (#7558, Cell Signaling Technology, Danvers, MA, USA) at 1:1000, and anti-α-tubulin (#T6199, Sigma-Aldrich, St. Louis, MO, USA) at 1:100,000 was performed to identify ABCB1, ABCG2, HDAC6, and tubulin as a positive control for Western blotting, as described previously [56]. The horseradish peroxidase-conjugated goat anti-mouse immunoglobulin G (IgG) and anti-rabbit IgG were used as secondary antibodies, and signals were detected as described previously [53].

### 4.5. Apoptosis Assays

The conventional annexin V–FITC and propidium iodide (PI) staining method was performed based on the method reported by Anderson et al. [70] to determine the apoptotic effect of citarinostat in human cancer cells overexpressing ABCB1 or ABCG2. Briefly, cells were treated with DMSO alone (control), or citarinostat alone or citarinostat in combination with tariquidar or Ko143 for 48 h before harvested by a series of washing plus centrifugation steps. Cells were subsequently resuspended in fluorescence-activated cell sorting (FACS) buffer containing 1.25 µg/mL annexin V–FITC (PharMingen) and 0.1 mg/mL PI and incubated for 15 min at room temperature. The labeled cells (10,000 per sample) were collected analyzed by FACScan (BD Biosciences) using the CellQuest software (Becton-Dickinson) as described previously [56].

### 4.6. Citarinostat Accumulation Assay and HPLC-MS/MS Analysis

The intracellular accumulation of citarinostat was quantified as described previously [71] with slight modification. In brief, 2 × 10^6^ cells were incubated with 10 μM of citarinostat in the presence or absence of 10 μM of tariquidar or Ko143 at 37 °C for 60 min. After washing twice with cold phosphate-buffered saline (PBS), cells were harvested and extracted with three volumes of methanol and stored at −20 °C overnight. After spinning down (10,000 rpm) at 4 °C for 30 min, the supernatants were first dried using the speed-vacuum-drying method and then redissolved with 50% methanol/0.1% formic acid followed by LC-MS/MS analysis. Cell contents were analyzed using ACQUITY UPLC-SRM/MS (ultra-performance liquid chromatography, selected reaction monitoring) analysis on a Waters BEH C18 Column (130Å, 1.7 µm, 1 × 100 mm, Waters Corporation, Milford, MA, USA) coupled with high capacity ion trap (HCT) ultra mass spectrometry (Bruker Daltonik GmbH, Bremen, Germany). Mobile phase A: water; B: acetonitrile, both containing 0.1% formic acid. A flow rate of 60 µL/min with a linear gradient was set as follows: started at 20% B for 270 s, and elevated to 95% B within 30 s; finally to 20% B within 108 s and then equilibrated for 192 s. Each sample was analyzed for 10 min, and the column temperature was maintained at 40 °C. Ion transition of citarinostat (parent ion m/z 468.4, fragment ion m/z 308.3) was monitored by selected reaction monitoring (SRM) in positive mode. The peak area of the MS2 fragment (m/z 308.3) was selected for quantitation and integrated with DataAnalysis 4.2 (Bruker Corporation, Billerica, MA, USA). The concentration of the citarinostat response curve was set, ranging from 50 fmol/μL to 50 pmol/μL, using cell lysate extracts as a background.

### 4.7. Docking of Citarinostat in the Substrate-Binding Pocket of Human ABCB1 and ABCG2

AutoDock Vina [72] was used to dock citarinostat to the atomic structures of ABCB1 (PDBID:6QEX) [58] and ABCG2 (PDB:5NJ3) [59] as previously described [73]. Proteins and ligands were prepared using the MGLtools software package (Scripps Research Institute) [74]. Analysis of the docked poses was performed using the Pymol molecular graphics system, Version 1.7 (Schrödinger, LLC, New York, NY, USA).

### 4.8. Quantification and Statistical Analysis

The cytotoxicity data are presented as mean ± standard error of the mean (S.E.M). The IC_50_ values are mean ± standard deviation (S.D.) calculated from at least three independent experiments. Curve plotting was performed using GraphPad Prism (La Jolla, CA, USA) software, and statistical analysis was performed using KaleidaGraph (Reading, PA, USA) software. The improvement in fit was analyzed by two-tailed Student’s t-test and labeled “statistically significant” if the probability, *p*, was less than 0.05.

## 5. Conclusions

**Figure 6:**
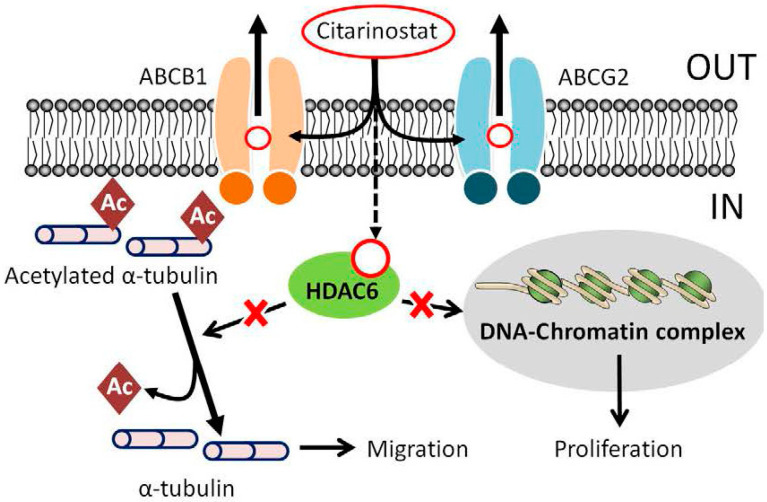
A schematic illustrating ABCB1- and ABCG2-mediated efflux of citarinostat in human multidrug-resistant cancer cells. The intracellular accumulation of citarinostat in ABCB1- and ABCG2-expressing cancer cells is significantly reduced due to the drug efflux function of ABCB1 (orange) and/or ABCG2 (blue). Consequently, the inhibitory effect of citarinostat on the activity of HDAC6 and its antiproliferative effects are both significantly decreased in drug resistant cancer cells expressing these transporters.

In summary, as shown in schematic illustration (Figure 6), our study revealed the effect of ABCB1 and ABCG2 on the pharmacological impact of citarinostat, which may play an important role in the development of resistance to citarinostat in cancer cells. Since synthetic inhibitors against ABCB1 and ABCG2 have mostly failed due to systemic toxicity, further investigation of the combination of citarinostat with *repurposed FDA*-*approved modulators of* ABCB1 and ABCG2 is warranted.

## Figures and Tables

**Table 1:** The effect of tariquidar and Ko143 on the cytotoxicity of citarinostat in ABCB1- or ABCG2-overexpressing cell lines.

Cell Line	Mean IC_50_ ± SD [μM] ^1^ (R.F ^2^)		
	Citarinostat	Citarinostat + Tariquidar	Citarinostat + Ko143
KB-3-1	3.98 ± 0.73 (1)	4.59 ± 0.67 (1)	N.D
KB-V-1	57.96 ± 5.56 (15)	3.01 ± 0.57 *** (1)	N.D
OVCAR-8	7.52 ± 1.10 (1)	7.46 ± 1.55 (1)	N.D
NCI-ADR-RES	84.15 ± 9.91 (11)	5.56 ± 1.10 *** (1)	N.D
S1	2.82 ± 0.40 (1)	N.D	2.75 ± 0.45 (1)
S1-M1-80	59.66 ± 10.32 (21)	N.D	2.94 ± 0.50 *** (1)
H460	7.46 ± 0.36 (1)	N.D	6.17 ± 0.53 * (1)
H460-MX20	23.70 ± 1.58 (3)	N.D	6.08 ± 0.36 *** (1)
pcDNA-HEK293	3.01 ± 0.44 (1)	2.18 ± 0.21 (1)	1.57 ± 0.20 ** (1)
MDR19-HEK293	33.83 ± 3.21 (11)	3.19 ± 0.21 *** (1)	N.D
R482-HEK293	20.11 ± 2.48 *** (7)	N.D	4.78 ± 0.37 *** (3)

Abbreviation: R.F, resistance factor. N.D, not determined. ^1^IC_50_ values are mean ± SD in the presence and absence of 1 μM tariquidar or 1 μM Ko143. The IC_50_ values were calculated from dose–response curves obtained from three independent experiments. ^2^RF values were calculated by dividing IC_50_ value of citarinostat in ABCB1- or ABCG2-expressing cells by IC_50_ value of citarinostat in respective parental cells. * *p* < 0.05; ** *p* < 0.01; *** *p* < 0.001.

## Data Availability

The data presented in this study are available on request from the corresponding author.

## Supplementary Materials

The following data are available online at https://www.mdpi.com/1422-0067/22/5/2592/s1, Figure S1: The effect of citarinostat on the protein expression of Ac-tub and HDAC6 in human epidermal KB-3-1 and KB-V-1 cancer cells, and human colon S1 and S1-M1-80 cancer cells.
