# Supplementary material for: Overexpression of Human ABCB1 and ABCG2 Reduces the Susceptibility of Cancer Cells to the Histone Deacetylase 6-Specific Inhibitor Citarinostat
Source: Int J Mol Sci. 2021 Mar 5;22(5):2592. doi: 10.3390/ijms22052592 (PMC7961520; doi:10.3390/ijms22052592)
Supplement: Supplementary file 1 [file ijms-22-02592-s001.pdf]

Supplementary Materials

Overexpression of Human ABCB1 and ABCG2 Reduces the Susceptibility of Cancer Cells to the Histone Deacetylase 6-Specific Inhibitor Citarinostat

Chung-Pu Wu , Cheng-Yu Hung, Sabrina Lusvarghi, Yen-Fu Chang, Sung-Han Hsiao, Yang-Hui Huang, Tai-Ho Hung, Jau-Song Yu, and Suresh. V. Ambudkar

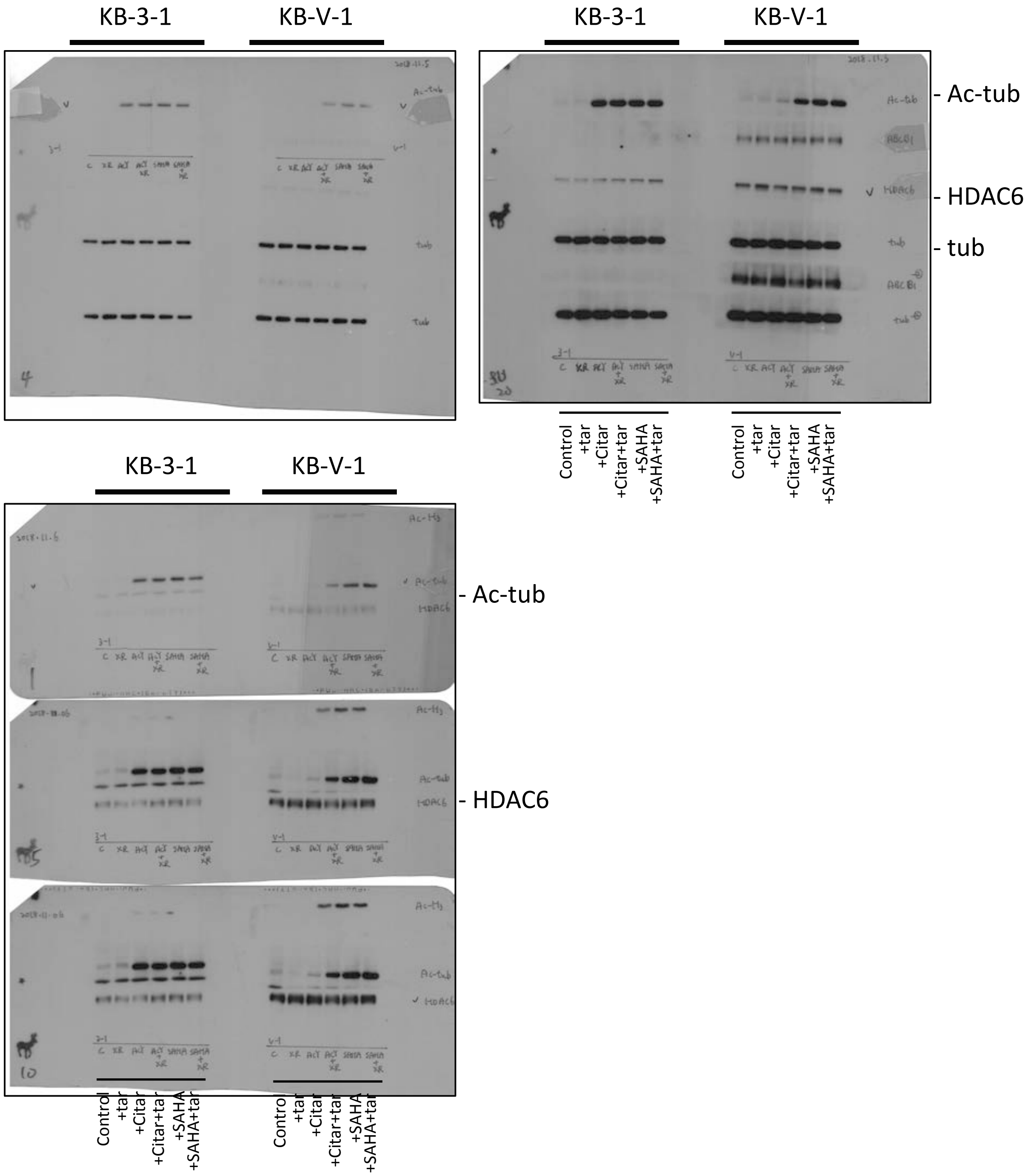

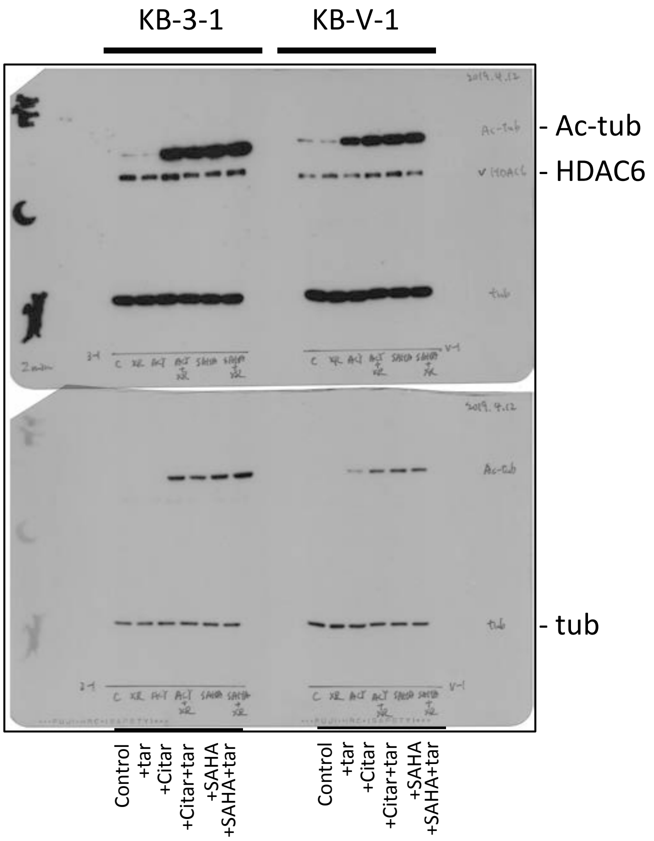
KB-3-1
KB-V-1
- Ac-tub
- HDAC6
- tub
Control
+tar
+Citar
+Citar+tar
+SAHA
+SAHA+tar
Control
+tar
+Citar
+Citar+tar
+SAHA
+SAHA+tar

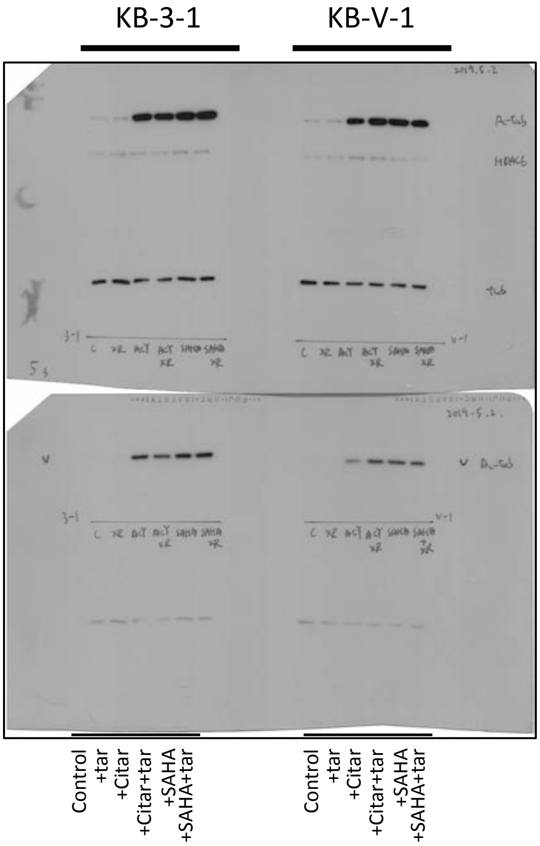
KB-3-1
KB-V-1
Control
+tar
+Citar
+Citar+tar
+SAHA
+SAHA+tar
Control
+tar
+Citar
+Citar+tar
+SAHA
+SAHA+tar

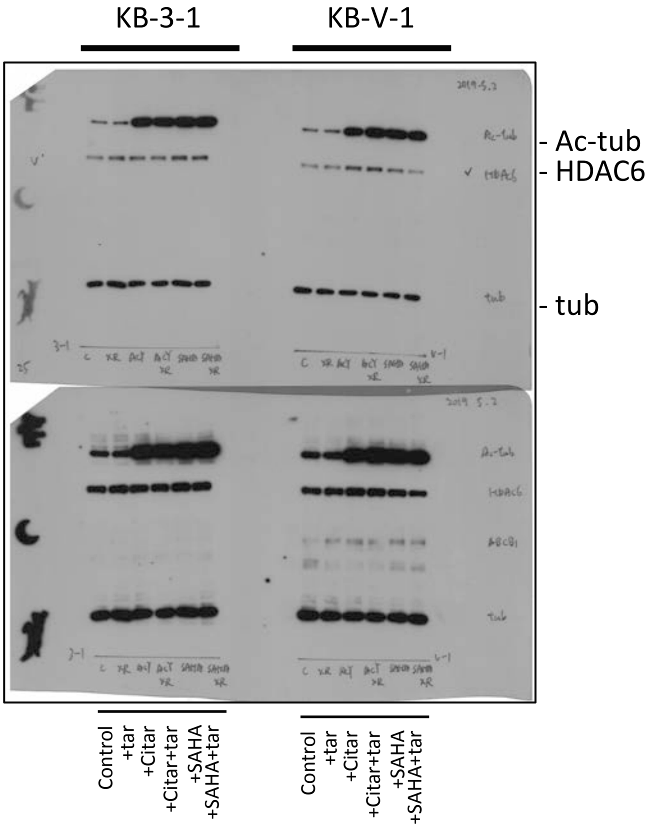
KB-3-1
KB-V-1
- Ac-tub
- HDAC6
- tub
Control
+tar
+Citar
+Citar+tar
+SAHA
+SAHA+tar
Control
+tar
+Citar
+Citar+tar
+SAHA
+SAHA+tar

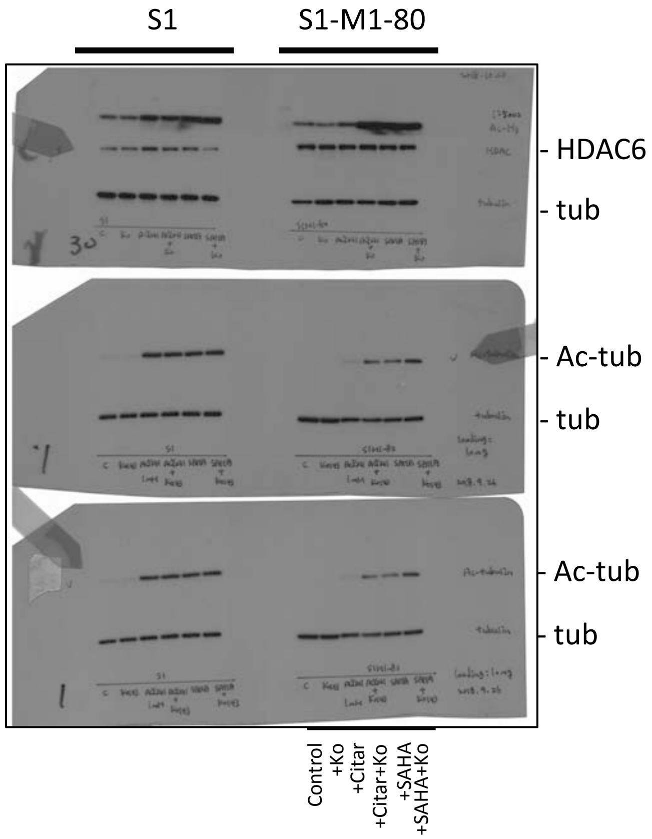
S1
S1-M1-80
HDAC6
tub
Ac-tub
tub
Ac-tub
tub
Control
+Ko
+Citar
+Citar+Ko
+SAHA
+SAHA+Ko

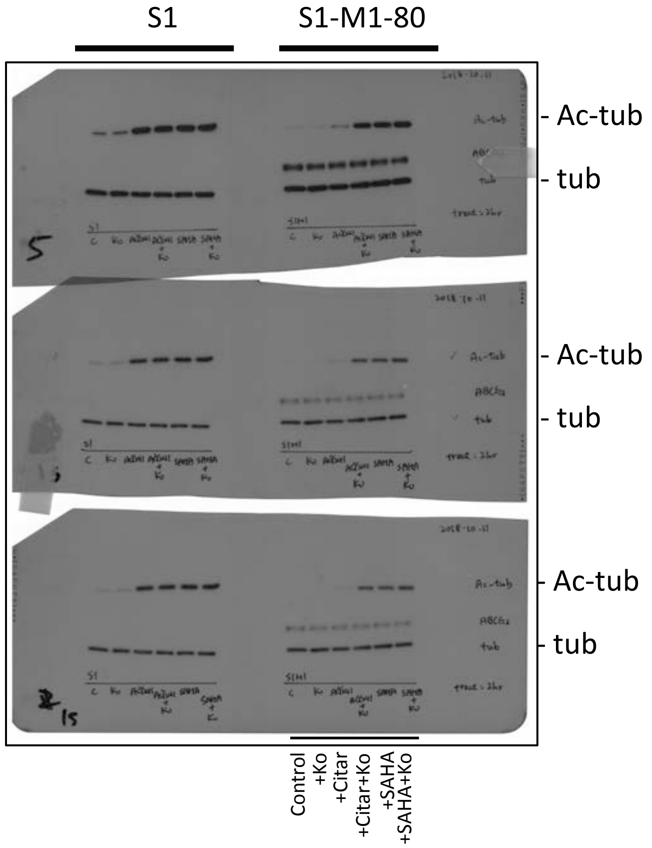
S1
S1-M1-80
Ac-tub
tub
Ac-tub
tub
Ac-tub
tub
Control
+Ko
+Citar
+Citar+Ko
+SAHA
+SAHA+Ko

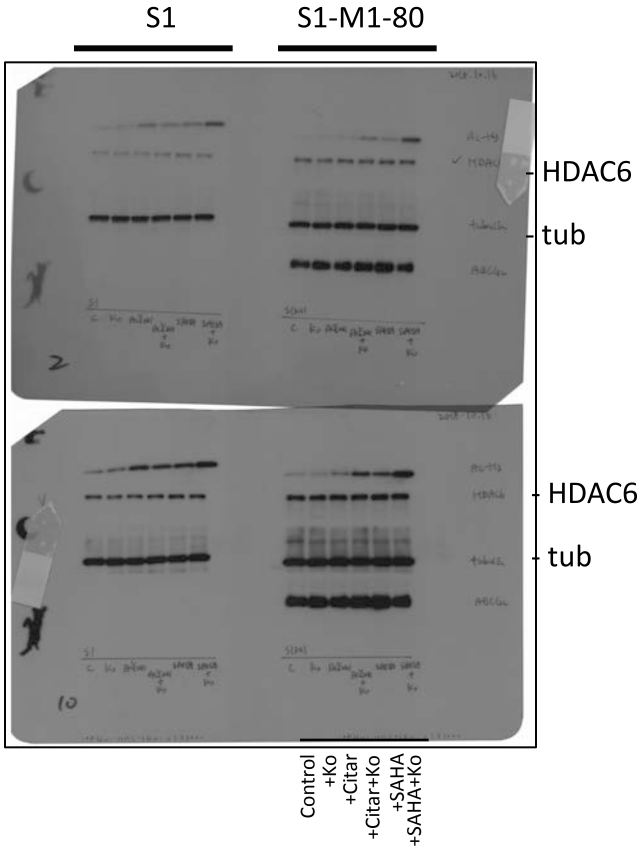
S1
S1-M1-80
HDAC6
tub
HDAC6
tub
Control
+Ko
+Citar
+Citar+Ko
+SAHA
+SAHA+Ko

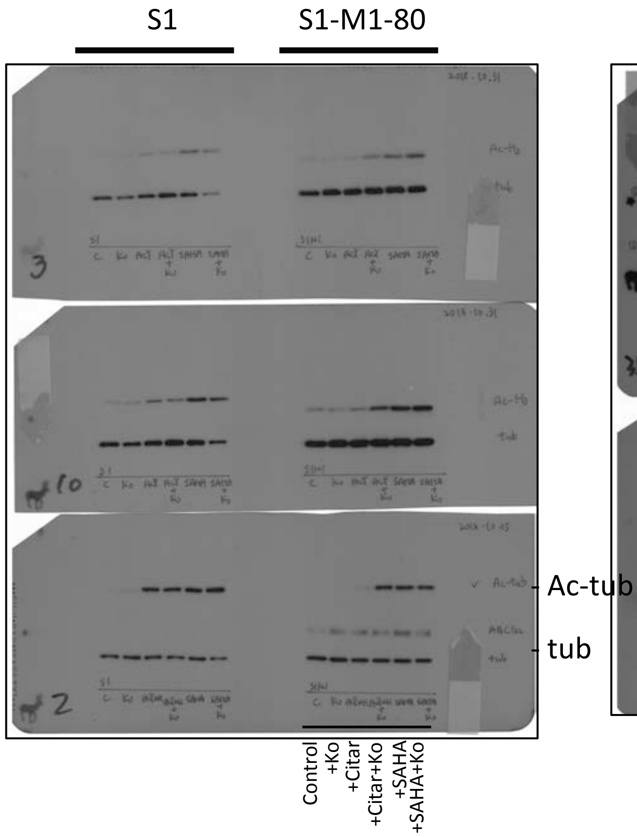

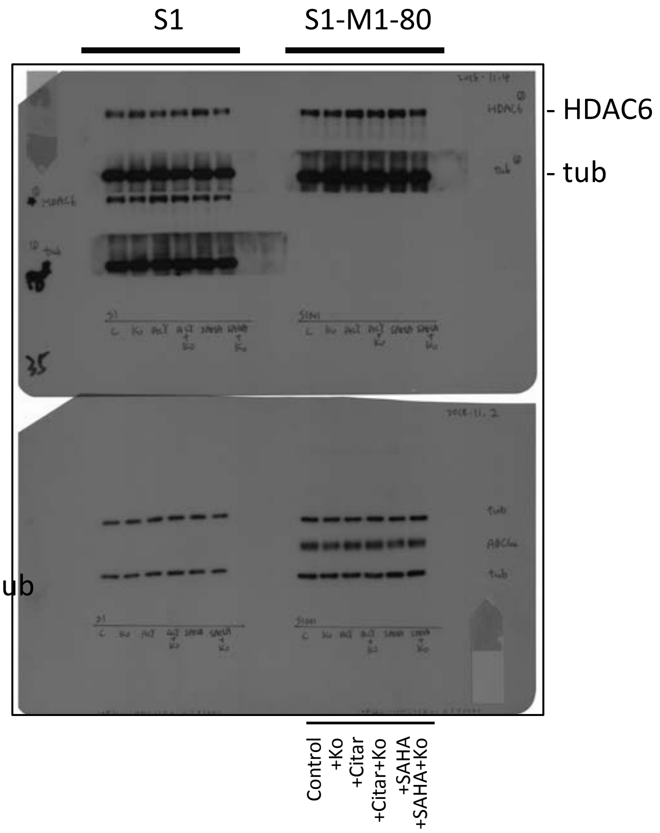

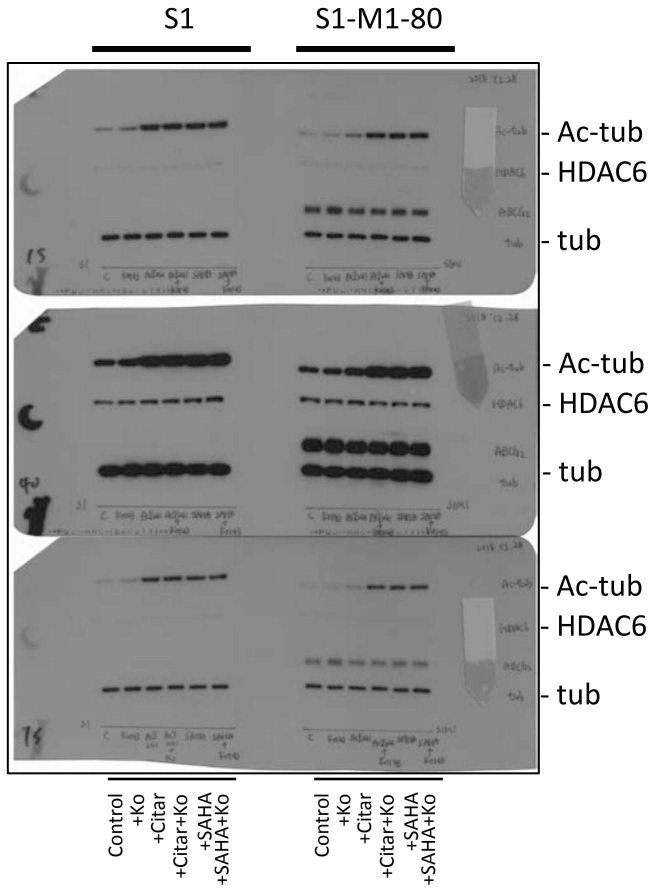

Figure S1: The effect of citarinostat on the protein expression of Ac-tub and HDAC6 in human epidermal KB-3-1 and KB-V-1 cancer cells, and human colon S1 and S1-M1-80 cancer cells.
